# PHF5A regulates the expression of the DOCK5 variant to promote HNSCC progression through p38 MAPK activation

**DOI:** 10.1186/s13062-023-00396-4

**Published:** 2023-07-12

**Authors:** Chao Liu, Guo Li, Siyuan Zheng, Li She, Shanhong Lu, Yunyun Wang, Donghai Huang, Xin Zhang, Lunquan Sun, Yong Liu, Yuanzheng Qiu

**Affiliations:** 1 Department of Otolaryngology Head and Neck Surgery, Xiangya Hospital, Central South University, 87 Xiangya Road, Changsha, Hunan 410008 China; 2 Otolaryngology Major Disease Research Key Laboratory of Hunan Province, Changsha, Hunan 410008 China; 3 Clinical Research Center for Pharyngolaryngeal Diseases and Voice Disorders in Hunan Province, Changsha, Hunan 410008 China; 4 National Clinical Research Center for Geriatric Disorders (Xiangya Hospital), Changsha, Hunan 410008 China; 5 Key Laboratory of Molecular Radiation Oncology Hunan Province, Changsha, Hunan 410008 China

**Keywords:** Head and neck squamous cell carcinoma, Alternative splicing, PHF5A, DOCK5

## Abstract

**Background:**

Previously, we identified an oncogenic splicing variant of DOCK5 in head and neck squamous cell carcinoma (HNSCC); however, the mechanism for the generation of this specific DOCK5 variant remains unknown. This study aims to explore the potential spliceosome genes involved in the production of the DOCK5 variant and validate its role in regulating the progression of HNSCC.

**Methods:**

The differentially expressed spliceosome genes involved in the DOCK5 variant were analysed in The Cancer Genome Atlas (TCGA), and the correlation between the DOCK5 variant and the potential spliceosome gene PHF5A was verified by qRT-PCR. The expression of PHF5A was detected in HNSCC cells, TCGA data and a separate primary tumour cohort. The functional role of PHF5A was examined using CCK-8, colony formation, cell scratch and Transwell invasion assays in vitro and validated in vivo in xenograft models of HNSCC. Western blot analysis was used to explore the potential mechanism of PHF5A in HNSCC.

**Results:**

PHF5A was one of the top upregulated spliceosome genes in TCGA HNSCC samples with highly expressed DOCK5 variants. Knockdown or overexpression of PHF5A in HNSCC cells correspondingly altered the level of the DOCK5 variant. PHF5A was highly expressed in tumour cells and tissues and correlated with a worse prognosis of HNSCC. Loss- and gain-of-function experiments demonstrated that PHF5A could promote the proliferation, migration and invasion of HNSCC cells in vitro and in vivo. Moreover, PHF5A inhibition reversed the oncogenic effect of the DOCK5 variant in HNSCC. Western blot analysis showed that PHF5A activated the p38 MAPK pathway, and inhibition of p38 MAPK further reversed the effect of PHF5A on the proliferation, migration and invasion of HNSCC cells.

**Conclusion:**

PHF5A regulates the alternative splicing of DOCK5 to promote HNSCC progression through p38 MAPK activation, which provides potential therapeutic implications for HNSCC patients.

**Supplementary Information:**

The online version contains supplementary material available at 10.1186/s13062-023-00396-4.

## Background

Head and neck cancer is the seventh most common cancer worldwide, and the main pathological type is head and neck squamous cell carcinoma (HNSCC) [1, 2]. Although treatments including surgery, radiochemotherapy, and immunotherapy have been substantially developed over the past several decades, the five-year survival rate of patients with advanced HNSCC remains relatively poor [3, 4]. The malignant traits of metastasis and recurrence severely restrict the therapeutic effect of HNSCC [5, 6]. Therefore, exploring the molecular mechanisms underlying the malignant progression of HNSCC may contribute to the development of novel targets for improved treatment.

Alternative splicing is a crucial process that enables a messenger RNA precursor to produce multiple spliced mRNAs, which increases the complexity of gene expression and protein diversity [7]. Alternative splicing is catalysed by the spliceosome, a multimegadalton protein-RNA complex comprised of five small nuclear ribonucleoproteins (snRNPs; U1, U2, U4, U5, and U6) and hundreds of related proteins [8]. Emerging evidence has demonstrated that the dysregulation of spliceosome genes is the main cause of aberrant splicing [9, 10]. Considering the importance of alternative splicing in gene regulation, numerous studies have thus revealed global dysregulation of splicing in various human diseases, including cancer [11]. For example, spliceosome genes that encode regulators of alternative splicing are usually overexpressed in cancer and lead to the generation of specific splicing variants that are crucial for cancer cell growth and survival [12, 13]. Alternative splicing can also regulate malignant biological behaviours of cancer, including proliferation, invasion, metastasis and radiochemoresistance [13, 14]. In HNSCC, we previously established the differential expression profile of alternative splicing through analysis of The Cancer Genome Atlas (TCGA) RNA sequencing data and validated that a novel DOCK5 splicing variant promoted the proliferation, migration and invasion of HNSCC cells [15]. However, the regulatory mechanism for the generation of this specific DOCK5 variant in HNSCC has yet to be elucidated.

In this study, we identified that PHD finger protein 5A (PHF5A), an important component of the SF3b complex in U2 snRNP, enhanced the production of the DOCK5 variant in HNSCC. Through loss- and gain-of-function experiments, PHF5A was confirmed to promote HNSCC cell proliferation, migration and invasion via the p38 MAPK pathway in vitro and in vivo. These results provide new insight into elucidating the role of PHF5A and DOCK5 variants in HNSCC tumorigenesis, which may be potential therapeutic targets for HNSCC patients.

## Results

### PHF5A promotes the expression of the DOCK5 variant in HNSCC

**Fig. 1:**
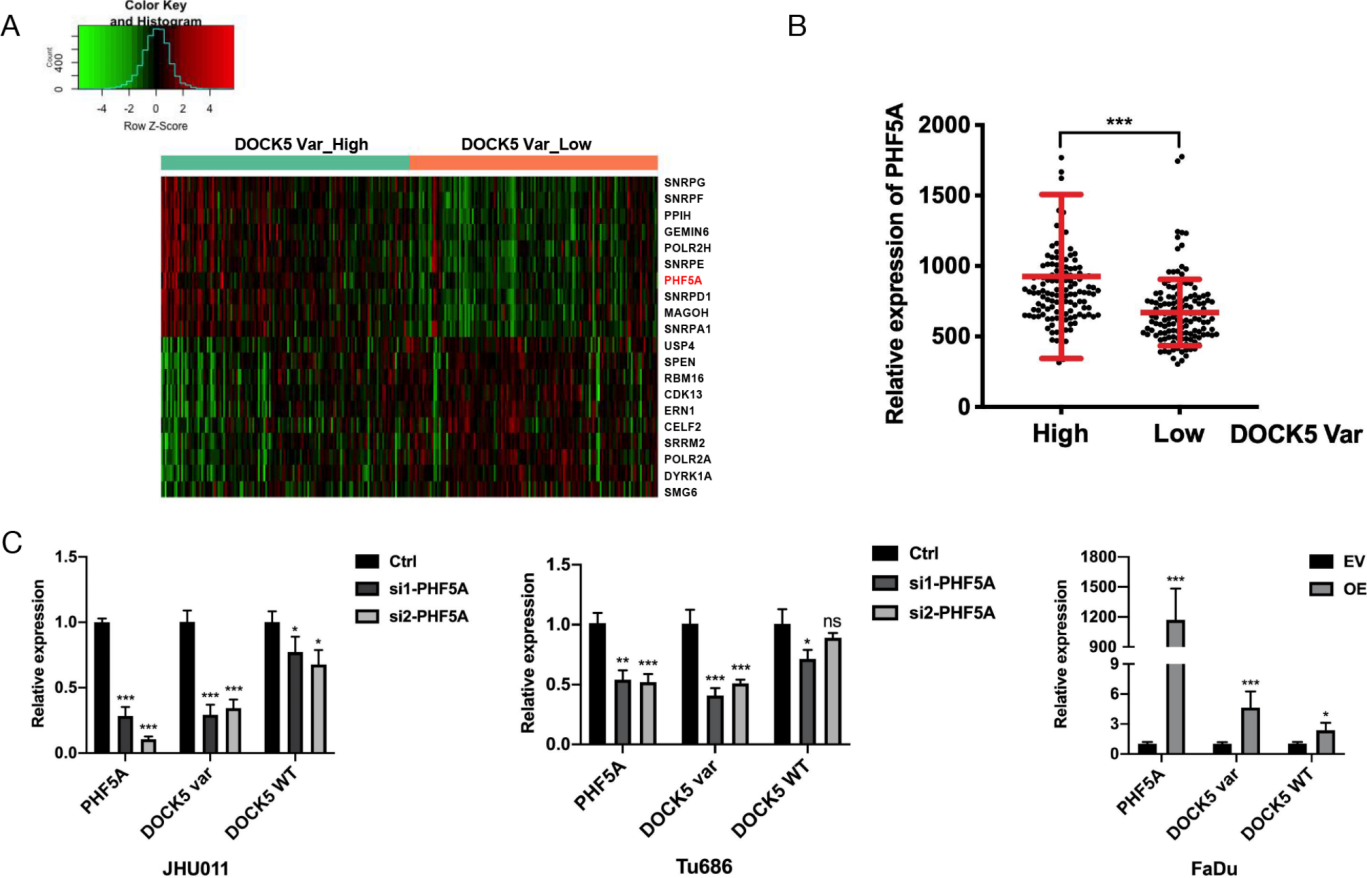
PHF5A promotes the expression of the DOCK5 variant in HNSCC **(A)** Top 10 upregulated and downregulated spliceosome genes between patients with higher and lower DOCK5 variant expression in TCGA HNSCC data. **(B)** The expression of PHF5A in groups of patients with high and low DOCK5 variant expression in TCGA HNSCC data. **(C)** The expression of PHF5A and DOCK5 variants following downregulation of PHF5A in JHU011 and Tu686 cells and upregulation of PHF5A in FaDu cells. ***: *P* < 0.001, **: *P* < 0.01, *: *P* < 0.05, ns: not significant. Ctrl indicates control; EV, empty vector group; OE, PHF5A overexpression group; WT, wild-type

Previously, we validated a novel DOCK5 splicing variant that promotes the progression of HNSCC [15]. To further explore the regulatory mechanism for the generation of this specific DOCK5 variant, we established the differential gene expression profile between high and low DOCK5 variant expression samples in TCGA HNSCC data [15]. Among the 319 spliceosome genes identified previously [16], PHF5A was one of the top significantly differentially expressed spliceosome gene (Fig. 1A) and was upregulated in high DOCK5 variant expression samples (Fig. 1B). Moreover, we downregulated the expression of PHF5A by transfecting two siRNAs targeting PHF5A into HNSCC JHU011 and Tu686 cells, upregulated the expression of PHF5A by stable transfection of PHF5A cDNA into HNSCC FaDu cells, and found that following downregulation or upregulation of PHF5A in HNSCC cells, the expression of the DOCK5 variant was also significantly decreased or increased correspondingly, with only a modest effect on DOCK5 wild-type gene expression (Fig. 1C). These results suggested that PHF5A promoted the expression of the DOCK5 variant in HNSCC.

### PHF5A is highly expressed in HNSCC cells and tissues

**Fig. 2:**
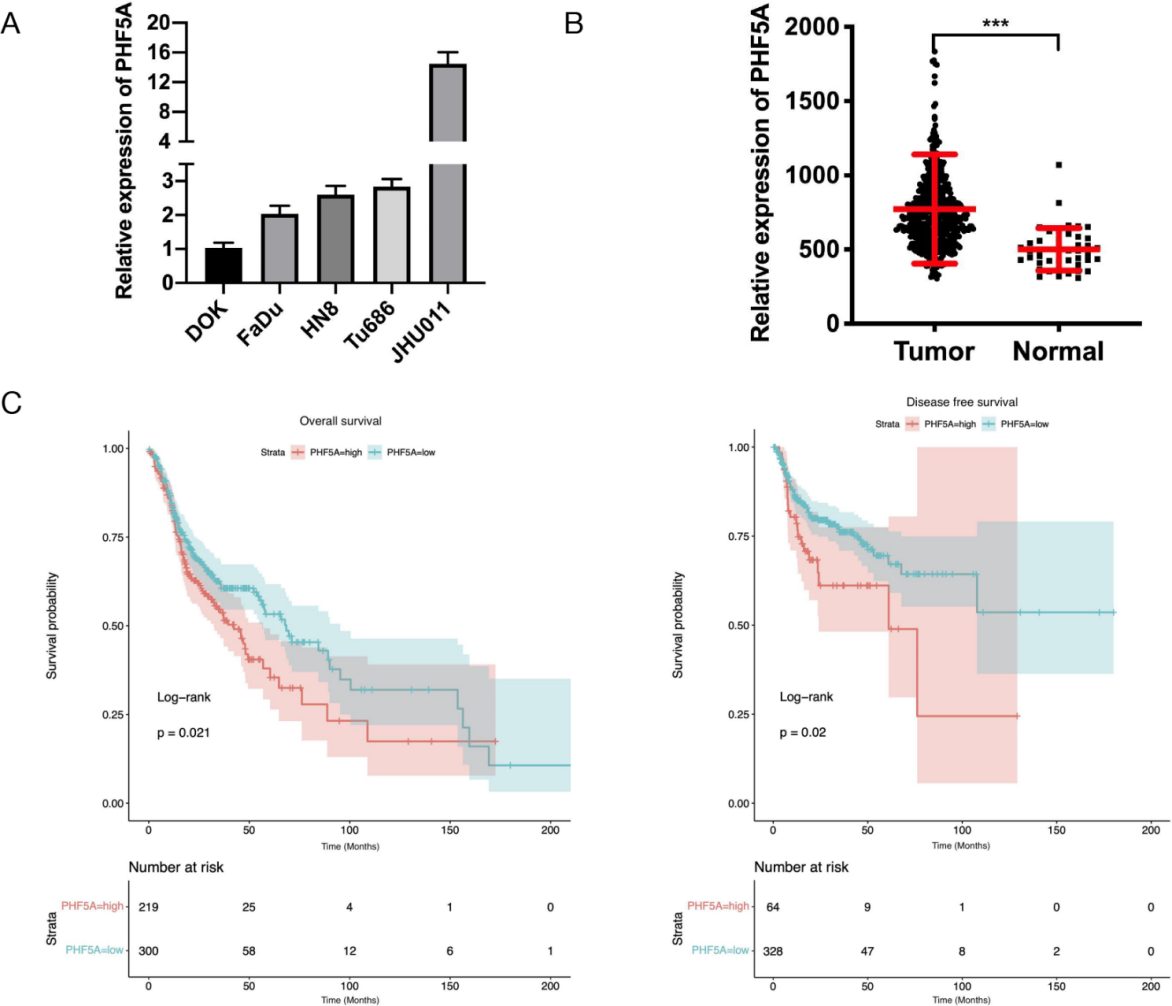
Expression of PHF5A in HNSCC cells and TCGA HNSCC data **(A)** Basic expression of PHF5A in DOK and HNSCC cells by qRT-PCR. **(B)** The expression of PHF5A in tumour and normal tissues in TCGA HNSCC data. **(C)** Overall survival and disease free survival in patients with high and low PHF5A expression in TCGA HNSCC data (best cut-off value determined by the R Survminer package)

qRT-PCR was used to examine the expression of PHF5A in HNSCC FaDu, HN8, Tu686, JHU011 cells and the precancerous lesions of the oral mucosa cell line DOK. The results demonstrated that compared with that in DOK cells, the expression of PHF5A was upregulated in HNSCC cells (Fig. 2A). In the TCGA data, the expression of PHF5A was higher than that in normal tissues (Fig. 2B), and higher expression of PHF5A predicted decreased overall survival and disease free survival in HNSCC patients (Fig. 2C). We also found that high PHF5A expression was associated with clinical parameters such as lymph node metastasis, smoking and alcohol consumption in TCGA data (Supplementary Table S2).

**Fig. 3:**
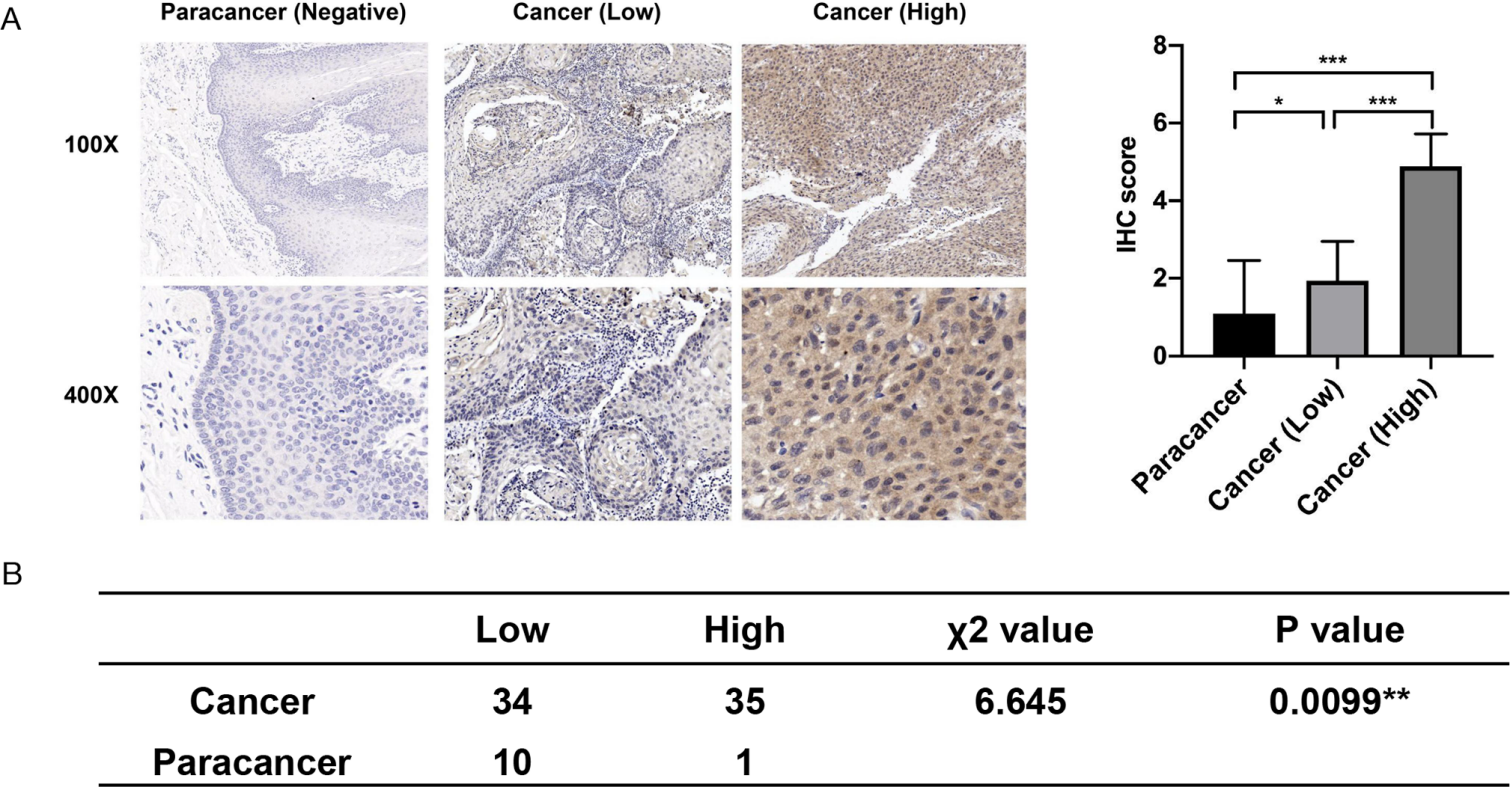
Expression of PHF5A in HNSCC tissues **(A)** Representative immunohistochemical staining for the PHF5A protein in HNSCC tissues and adjacent paracancerous tissues. **(B)** The expression of PHF5A in HNSCC tissues and adjacent paracancerous tissues

To further confirm the expression of PHF5A in HNSCC, we performed immunohistochemistry staining to detect the expression of PHF5A in a validation cohort of 69 primary HNSCC and 11 adjacent paracancerous tissues. The results showed that PHF5A was also overexpressed in HNSCC tissues compared with adjacent paracancerous tissues (Fig. 3A, B). As shown in Supplementary Table S3, PHF5A overexpression was significantly associated with primary tumour sites (P = 0.0171), T classifications (P = 0.0115), and clinical stages (P = 0.0374) in the HNSCC validation cohort. All these results indicated that PHF5A expression may be closely related to the progression of HNSCC.

### PHF5A promotes the proliferation of HNSCC

**Fig. 4:**
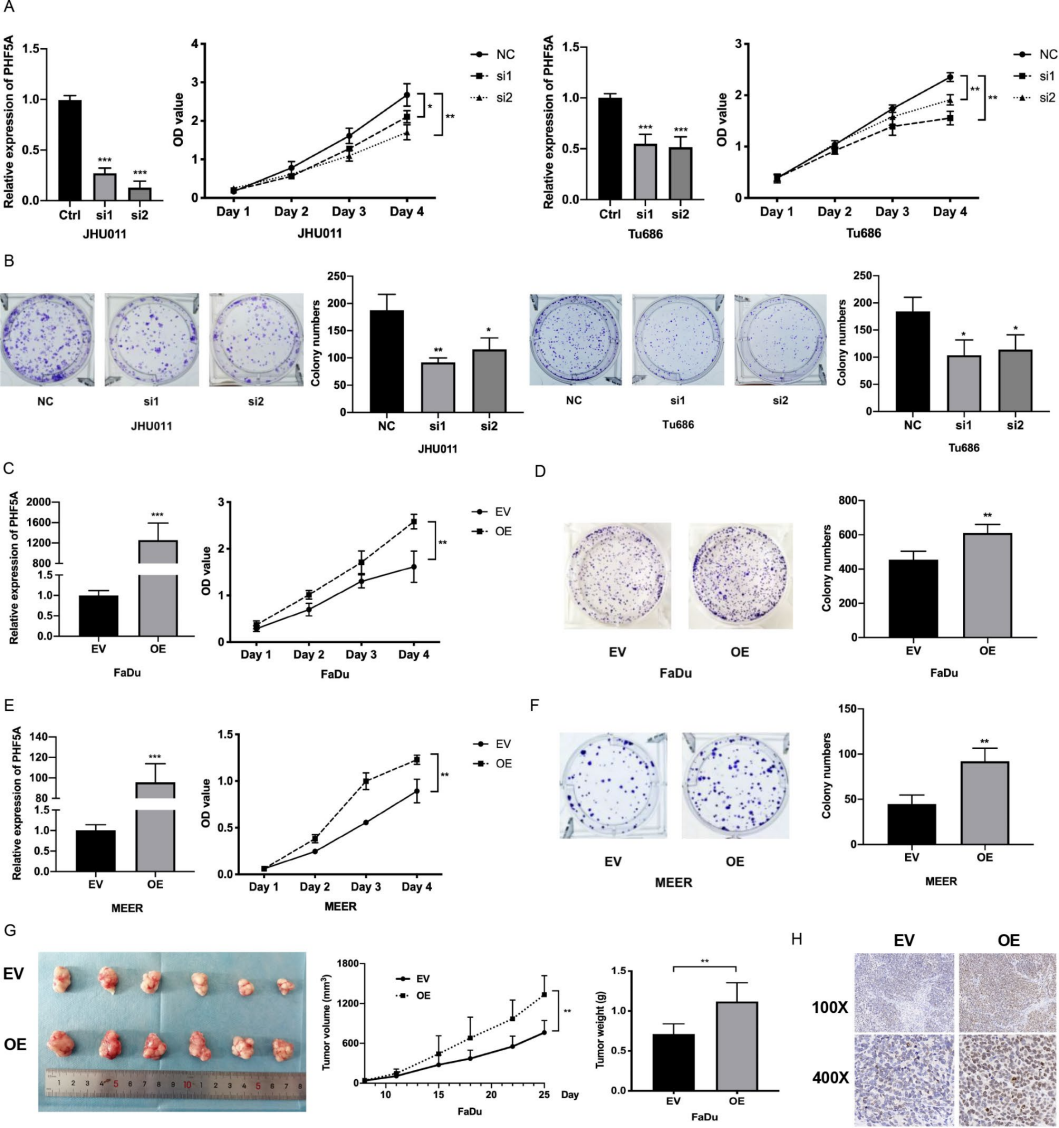
PHF5A promotes the proliferation of HNSCC **(A)** CCK-8 assays showed the proliferation of cancer cells after knockdown of PHF5A expression by two siRNAs in JHU011 and Tu686 cells. **(B)** Colony formation assays showed that colony formation was largely inhibited after knockdown of PHF5A expression in JHU011 and Tu686 cells. **(C, D)** CCK-8 **(C)** and colony formation assays **(D)** demonstrated that proliferation and colony formation were enhanced following overexpression of PHF5A in FaDu cells. **(E, F)** After overexpression of PHF5A in mouse MEER cells, CCK-8 **(E)** and colony formation assays **(F)** demonstrated that cell proliferation and colony formation were enhanced. **(G)** The final gross tumours, tumour volume and tumour weight are shown in the subcutaneous xenograft mouse model. **(H)** Representative immunohistochemical staining for the Ki-67 protein in the tumours of the subcutaneous xenograft mouse model. ***: *P* < 0.001, **: *P* < 0.01, *: *P* < 0.05. NC, negative control; EV, empty vector group; OE, PHF5A overexpression group

To investigate the functional role of PHF5A in HNSCC, we selected JHU011 and Tu686 cells with the highest expression of PHF5A for knockdown assays, and FaDu cells with the lowest expression of PHF5A were used for knock-in assays (Fig. 2A). The CCK-8 assay showed that following knockdown of PHF5A expression with two siRNAs, growth was significantly inhibited in both JHU011 and Tu686 cells (Fig. 4A). Colony formation assays demonstrated that smaller and fewer colonies were observed in the PHF5A knockdown group (Fig. 4B). For gain-of-function assays, CCK-8 and colony formation assays revealed that PHF5A overexpression promoted the growth and colony formation of FaDu cells (Fig. 4C, D). Moreover, we transfected mouse Phf5a cDNA into mouse HNSCC MEER cells and found that upregulation of PHF5A enhanced the proliferative ability of MEER cells (Fig. 4E, F). Furthermore, we established a subcutaneous xenograft mouse model of HNSCC using FaDu cells infected with empty vector (EV) and PHF5A overexpression (OE) plasmids, which showed that the tumours in the PHF5A overexpression group grew faster and weighed more than those in the empty vector group (Fig. 4G). Immunohistochemical staining showed that the expression of cell proliferation marker Ki-67 was also upregulated in PHF5A overexpressed xenografts (Fig. 4H). These in vitro and in vivo data revealed that PHF5A promoted the proliferation of HNSCC.

### PHF5A promotes the migration and invasion of HNSCC

**Fig. 5:**
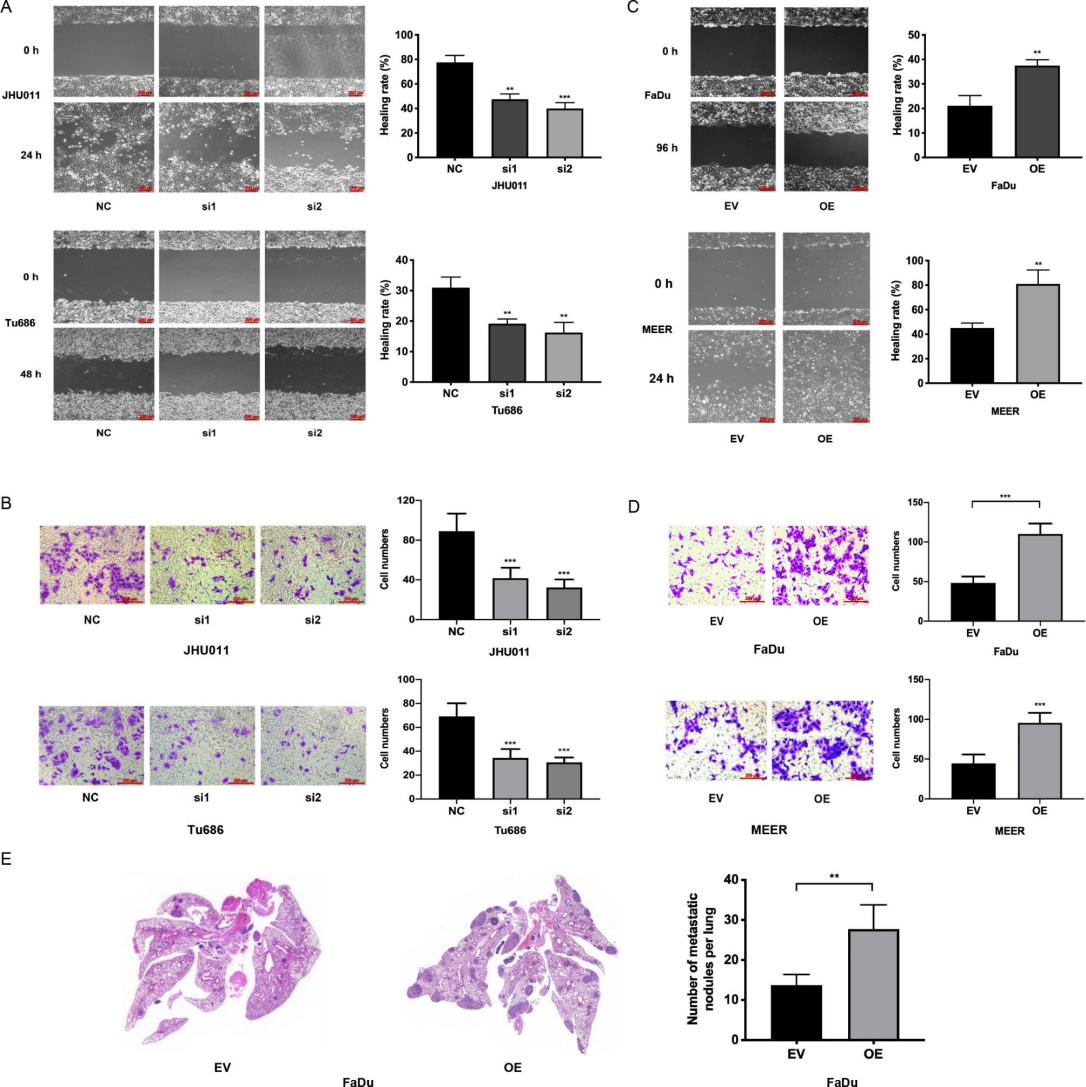
PHF5A promotes the migration and invasion of HNSCC **(A)** Cell scratch assays showed the migration of cancer cells after knockdown of PHF5A in JHU011 and Tu686 cells. **(B)** Transwell invasion assays showed that invasion was largely inhibited after knockdown of PHF5A in JHU011 and Tu686 cells. **(C, D)** Cell scratch assays **(C)** and Transwell invasion assays **(D)** demonstrated that migration and invasion were enhanced following overexpression of PHF5A in FaDu and MEER cells. **(E)** Representative images of macroscopic lung metastatic nodules were photographed, and the numbers of lung nodules were calculated in a metastasis mouse model (n = 4). ***: *P* < 0.001, **: *P* < 0.01. EV, empty vector group; OE, PHF5A overexpression group

To elucidate whether PHF5A affected the metastatic phenotype of HNSCC, we performed cell scratch and Transwell invasion assays to assess cell migration and invasion in vitro. As shown in Fig. 5A, the cell scratch assay demonstrated that knockdown of PHF5A inhibited the migration of both JHU011 and Tu686 cells. Transwell invasion assays revealed that the number of invading cells was decreased significantly by the downregulation of PHF5A in both JHU011 and Tu686 cells (Fig. 5B). Correspondingly, overexpression of PHF5A in FaDu and MEER cells significantly augmented cell migration and invasion (Fig. 5C, D). As these HNSCC cell lines barely develop metastatic lesions from subcutaneous xenografts, FaDu cells were injected via mouse tail veins to generate distant organ metastatic lesions. The in vivo metastasis model showed that PHF5A enhanced the formation of lung metastatic lesions of FaDu cells, with the number of lung metastatic nodules being increased in FaDu cells with PHF5A overexpression (Fig. 5E). Taken together, these results revealed that PHF5A promoted the migration and invasion of HNSCC.

### PHF5A inhibition reverses the effect of the DOCK5 variant in HNSCC

**Fig. 6:**
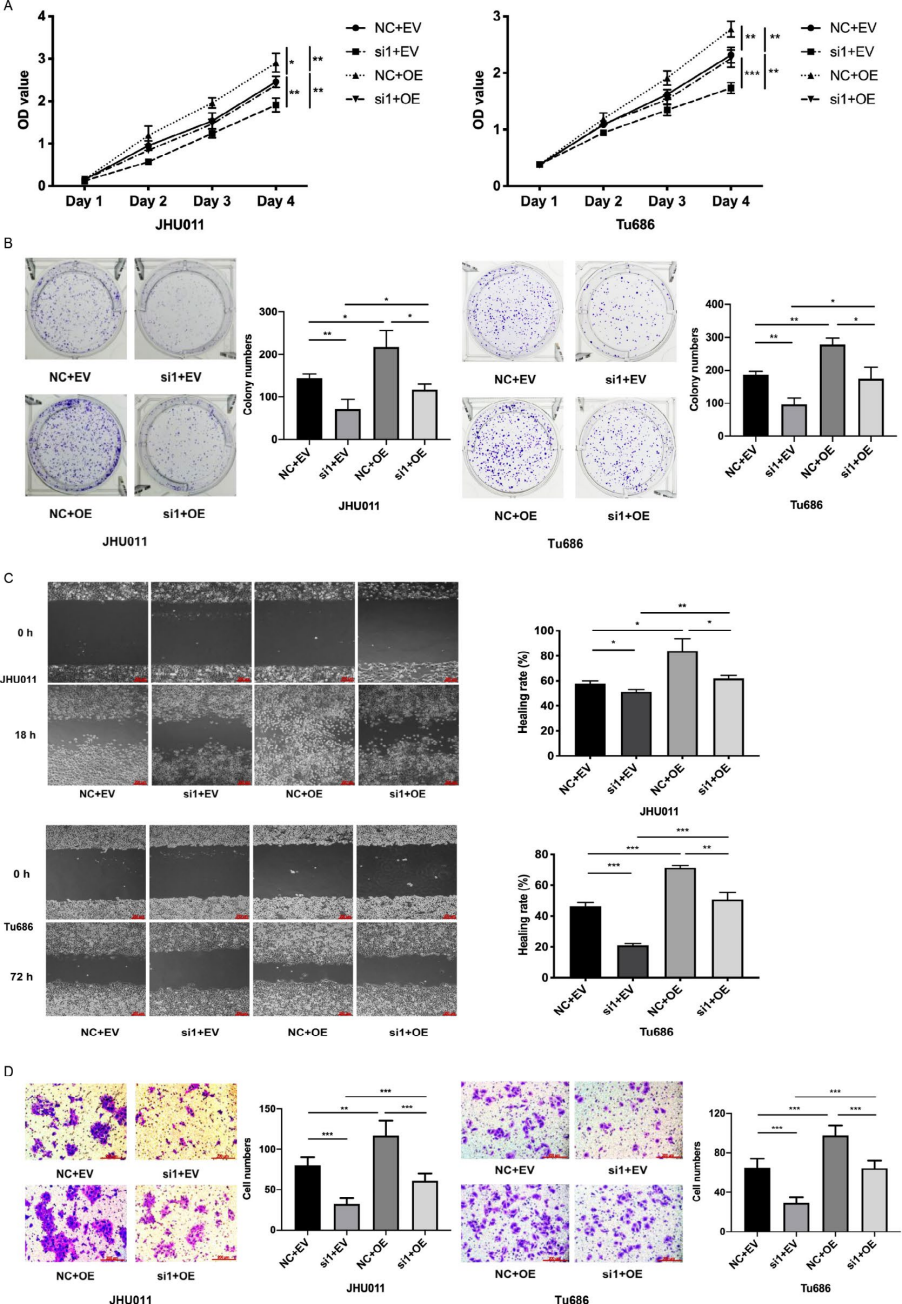
PHF5A inhibition reverses the effect of the DOCK5 variant in HNSCC **(A)** CCK-8 assays showed that PHF5A inhibition reversed the pro-proliferative effect of the DOCK5 variant in JHU011 and Tu686 cells. **(B)** Colony formation assays showed that PHF5A inhibition reversed the pro-colony formation effect of the DOCK5 variant in JHU011 and Tu686 cells. **(C)** Cell scratch assays showed that PHF5A inhibition reversed the pro-migratory effect of the DOCK5 variant in JHU011 and Tu686 cells. **(D)** Transwell invasion assays showed that PHF5A inhibition reversed the pro-invasive effect of the DOCK5 variant in JHU011 and Tu686 cells. NC, negative control; si1, PHF5A siRNA1 group; EV, empty vector group; OE, DOCK5 variant overexpression group

Based on our previous study and data, we confirmed that both PHF5A and DOCK5 variants promoted HNSCC progression and that PHF5A could enhance the expression of DOCK5 variants in HNSCC. To further investigate whether PHF5A regulates the expression of the DOCK5 variant to promote the progression of HNSCC, we performed rescue experiments by transfecting JHU011 and Tu686 cells with both PHF5A siRNA and DOCK5 variant overexpression plasmid. CCK-8 and colony formation assays showed that PHF5A inhibition reversed the effect of DOCK5 variant-mediated HNSCC proliferation (Fig. 6A, B). Moreover, cell scratch and Transwell invasion assays demonstrated that knockdown of PHF5A mitigated the enhancement of migration and invasion caused by the DOCK5 variant in JHU011 and Tu686 cells (Fig. 6C, D). Therefore, rescue experiments clearly indicated that PHF5A inhibition could reverse the effect of the DOCK5 variant in HNSCC, which suggested that PHF5A regulated the expression of the DOCK5 variant to promote the progression of HNSCC.

### PHF5A activates the p38 MAPK pathway in HNSCC

**Fig. 7:**
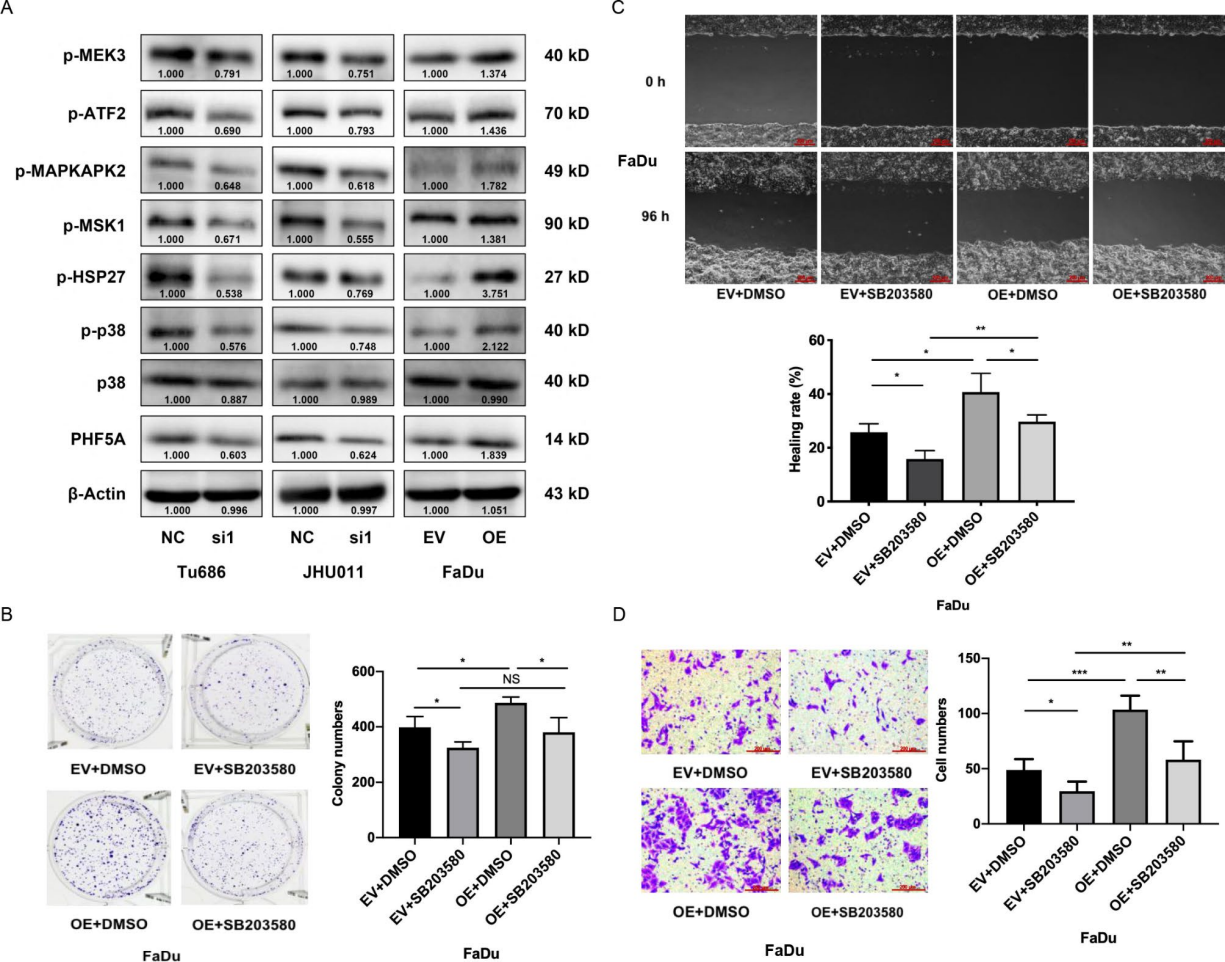
PHF5A activates the p38 MAPK pathway in HNSCC **(A)** The expression of PHF5A, p38, p-p38 and the downstream targets p-HSP27, p-MSK1, p-MAPKAPK2, p-ATF2 and p-MEK3 in the p38 MAPK pathway was assessed by Western blot following knockdown and overexpression of PHF5A in HNSCC cells. **(B)** Colony formation assays showed that p38 MAPK inhibition with SB203580 reversed the pro-colony formation effect of PHF5A in FaDu cells. **(C)** Cell scratch assays showed that SB203580 reversed the pro-migratory effect of PHF5A in FaDu cells. **(D)** Transwell invasion assays showed that SB203580 reversed the pro-invasive effect of PHF5A in FaDu cells. NC, negative control; si1, PHF5A siRNA1 group; EV, empty vector group; OE, PHF5A overexpression group

Previously, we found that the DOCK5 variant could activate the p38 MAPK pathway in HNSCC [15], so we hypothesized that PHF5A might be involved in the p38 MAPK pathway to promote HNSCC progression. Western blot results demonstrated that following knockdown of PHF5A expression in Tu686 and JHU011 cells, the expression levels of p-p38 and downstream targets p-HSP27, p-MSK1, p-MAPKAPK2, p-ATF2 and p-MEK3 all decreased, while total p38 was almost unchanged (Fig. 7A). Upregulation of PHF5A in FaDu cells increased the expression of p-p38, p-HSP27, p-MSK1, p-MAPKAPK2, p-ATF2 and p-MEK3 (Fig. 7A). For the subcutaneous xenografts, the expression of p-p38, p-MAPKAPK2 and p-MEK3 was also increased in the PHF5A overexpression group (Supplementary Fig. S1). To further validate the interaction between PHF5A and the p38 MAPK pathway, we used 10 µM SB203580 (a p38 MAPK specific inhibitor) to inactive p38 MAPK in FaDu cells [17]. Following the p38 MAPK pathway inhibited by SB203580 in FaDu cells (Supplementary Fig. S2), colony formation assays showed that inactivation of the p38 MAPK pathway significantly inhibited the pro-proliferative effect caused by PHF5A (Fig. 7B). Cell scratch and Transwell invasion assays demonstrated that inhibition of p38 MAPK could also attenuate the migratory and invasive ability triggered by PHF5A in FaDu cells (Fig. 7C, D). These data indicate that PHF5A activated the p38 MAPK pathway in HNSCC.

## Discussion

Elucidating the molecular mechanisms involved in the initiation and progression of HNSCC is beneficial to the development of novel therapeutic strategies for patients with HNSCC. Alternative splicing is closely associated with tumour occurrence and progression, and our previous study characterized the landscape of alternative splicing in HNSCC, in which a novel DOCK5 variant was confirmed to play an oncogenic role in HNSCC cell proliferation, migration, and invasion [15]. The present study further described the regulatory mechanism of the DOCK5 variant in HNSCC and found that the spliceosome gene PHF5A enhanced the generation of the DOCK5 variant to promote the progression of HNSCC through p38 MAPK activation.

Alternative splicing is an important mechanism for the regulation of gene expression and protein diversity and is carried out by the spliceosome, which consists of several snRNPs and over 300 different proteins in humans [18]. Multiple spliceosome genes have also been reported to drive carcinogenesis. For example, the spliceosome gene SRSF1 mediates tumour cell invasion and maintenance of stem cell properties by controlling Kras splicing in colorectal cancer [19], and SNRPA1 can regulate alternative splicing of PLEC and ERRFI1 to enhance metastatic lung colonization and cancer cell invasion in breast cancer [20]. In HNSCC, we previously determined that CPSF1 promotes aberrant splicing of cancer-associated genes and induces HNSCC cell proliferation and tumorigenicity [16]. In this study, by analysing the differentially expressed genes between patients with high and low DOCK5 variant expression in TCGA HNSCC data, we focused on the spliceosome gene PHF5A in regulating the production of DOCK5 variants in HNSCC, which was further validated by gene knockdown and knock-in assays. As for the other differentially expressed spliceosome genes, more studies were needed to investigate the potential functions.

PHF5A belongs to the superfamily of PHD-finger genes, which encode a subunit of the SF3b protein complex [21]. SF3b, together with SF3a and a 12S RNA unit, forms the U2 snRNP, an essential component of the spliceosome and is responsible for alternative splicing in all eukaryotes [22]. PHF5A exerts multiple biological functions, such as the regulation of embryo formation and tissue morphogenesis, DNA repair, chromatin remodelling, cell pluripotency and differentiation [23–25]. With regard to tumours, PHF5A is overexpressed in lung cancer, breast cancer, colorectal cancer and hepatocellular carcinoma [26–29] and promotes malignant tumour biological progression associated with the NF-κB pathway [29], RhoA/ROCK pathway [30], and activation of HDAC8 [31]. In particular, in colorectal cancer, PHF5A acetylation strengthened the interaction among U2 snRNPs and enhanced the alternative splicing-mediated upregulation of KDM3A to promote colorectal cancer tumorigenesis [32]. PHF5A could also promote colorectal cancer progression by alternative splicing of TEAD2 [28]. In lung cancer, PHF5A promoted cancer cell proliferation, invasion and migration by inducing genome-wide alternative splicing events [33]. In breast cancer, PHF5A is essential for cancer cell proliferation, migration, and tumour formation in part through alternative splicing of FASTK [27]. In glioblastoma, PHF5A facilitated the recognition of exons with unusual C-rich 3’ splice sites in multiple essential genes and enhanced alternative splicing to maintain glioblastoma stem cell expansion [34]. Thus, it was proposed that PHF5A participated in alternative splicing and played a critical role in cancer development. However, the biological function of PHF5A in HNSCC is still a mystery and needs to be further explored.

In this study, we identified PHF5A as the splicing factor for the DOCK5 variant in HNSCC and showed that PHF5A was highly expressed in HNSCC tissues, positively associated with T classifications and clinical stages in our clinical data, and related to lymph node metastasis and unfavourable prognosis in TCGA HNSCC data, which was consistent with previous findings in other types of cancer [26, 27]. As expected, in vitro and in vivo studies demonstrated that PHF5A not only increased tumour growth but also significantly strengthened the migratory and invasive capacity of HNSCC. Furthermore, we verified that PHF5A inhibition impaired the effect of the DOCK5 variant in HNSCC through a reverse experiment. Therefore, we concluded that PHF5A regulates the expression of the DOCK5 variant to promote the progression of HNSCC. Additional investigations are needed to clarify the exact mechanism by which PHF5A regulates the alternative splicing of DOCK5 in HNSCC.

MAPK pathway activity plays a key role in HNSCC progression [35], and our previous study revealed that the DOCK5 variant was associated with the p38 MAPK pathway [15]. Given the role of PHF5A in the generation of DOCK5 variants, this study demonstrated that PHF5A activated the p38 MAPK pathway in HNSCC, with increases in p-p38, p-HSP27, p-MSK1, p-MAPKAPK2, p-ATF2 and p-MEK3. These effects were further validated by inhibition of p38 MAPK with SB203580, while the pro-proliferative, migratory and invasive effects caused by PHF5A were largely attenuated. These results showed that PHF5A activated the p38 MAPK pathway in HNSCC, which enriched the theoretical mechanistic study of PHF5A in regulating cancer progression.

## Conclusion

The current study demonstrated that PHF5A regulated the expression of the DOCK5 variant to promote the proliferation, migration, and invasion of HNSCC through p38 MAPK activation. These data provide a novel perspective for understanding the malignant progression of HNSCC and might have significant implications for the development of novel targeted therapies for HNSCC patients.

## Methods

### Cell culture

The human HNSCC cell lines HN8 and JHU011 and the mouse HNSCC cell line MEER were kindly gifted by Dr Joseph Califano (University of California San Diego, USA). Tu686 was kindly provided by Dr Zhuo G. Chen (Emory University, USA). The FaDu cell line was purchased from ATCC. The precancerous lesions of the oral mucosa cell line DOK were obtained from The Cell Bank of Type Culture Collection of Chinese Academy of Sciences. These cells were cultured in appropriate medium (DMEM, DMEM/F12, or RPMI 1640; Sigma-Aldrich, St. Louis, MO, USA) with 10% foetal bovine serum (FBS; Gibco, Gaithersburg, MD, USA) and 1% penicillin-streptomycin (Gibco) at 37 °C under an atmosphere of 5% CO_2_. The cell lines FaDu and Tu686 were characterized using short tandem repeat (STR) profiling, and JHU011 and HN8 were authenticated previously [15, 36]. All experiments were conducted with mycoplasma-free cells.

### Patient samples

Primary HNSCC (n = 69) and adjacent paracancerous tissues (n = 11) were obtained from the Department of Otolaryngology Head and Neck Surgery, Xiangya Hospital, Central South University, Changsha, China. None of these patients had previously received neoadjuvant chemotherapy or radiotherapy. The study was performed in accordance with the Declaration of Helsinki and approved by the Research Ethics Committee of Xiangya Hospital, Central South University (No. 202,009,484). Informed consent was obtained from all patients prior to the clinical samples being collected.

### Transient transfection

Two siRNAs targeting PHF5A (Cat. No. stB0014349) and negative control (NC) siRNA were synthesized by Ribo Biotech (RiboBio, Guangzhou, China) and transfected into HNSCC cells with a riboFECT™ CP Transfection Kit (RiboBio) according to the manufacturer’s instructions. The overexpression plasmids for DOCK5 variant (OE; EX-Y5474-Lv201), mouse Phf5a (EX-Mm09732-Lv201), and the empty vector (EV; EX-NEG-Lv201) were obtained from GeneCopoeia, Inc. (Rockville, MD, USA) and transfected into cancer cells using FuGENE HD Transfection Reagent (Promega, Madison, WI, USA).

### Stable transfection

The overexpression plasmid for human PHF5A (EX-V1039-Lv201) was obtained from GeneCopoeia, Inc., which was used to generate lentiviral particles with EndoFectin-Lenti™ and TiterBoost™ reagents (GeneCopoeia, Inc.) following the standardized protocol. FaDu cells were infected with these specific or control lentiviruses and selected with 2 µg/ml of puromycin (Sigma-Aldrich) to establish stably transfected cells.

### Quantitative real-time PCR

Total RNA was isolated from cells with TRIzol reagent (Invitrogen, Carlsbad, CA, USA). The reverse transcription and detection of mRNA was carried out with the SureScript™ First-Strand cDNA Synthesis Kit and BlazeTaq™ SYBR Green qPCR Mix (GeneCopoeia, Inc.). PCR quantification was performed using the 2^−ΔΔCT^ method and normalization to GAPDH. The sequences of related primers are listed in Supplementary Table S1.

### Cell proliferation, migration and invasion assays

Cell proliferation was determined by CCK-8 and colony formation assays as we described previously [15, 36, 37]. A cell scratch assay was used to evaluate cell migration, in which a scratch was created with a 200 µl plastic pipette tip when the cells reached almost full confluence, and the cells were cultured in serum-free medium for 18–96 h to be photographed. Cell invasion was detected with Transwell Corning BioCoat Matrigel Invasion chambers (Corning, Inc., Corning, NY, USA) according to the manufacturer’s instructions. All experiments were repeated three or more times.

### Western blot analysis

Total proteins from cells or tissues were extracted using RIPA lysis buffer, separated by 10% SDS-PAGE gels, and then transferred onto polyvinylidene fluoride (PVDF) membranes (Millipore, Billerica, MA, USA). Then, the membranes were blocked with 5% BSA and incubated with the relevant primary antibody followed by an appropriate secondary antibody. Primary antibodies against p38, p-p38, p-HSP27, p-MSK1, and p-MAPKAPK2 were purchased from Cell Signaling Technology (Danvers, MA, USA). The antibodies against of p-ATF2 and p-MEK3 were obtained from Abcam (Waltham, MA, USA), and the PHF5A antibody was provided by Novus Biologicals (Littleton, CO, USA). Anti-β-Actin (Cell Signaling Technology) was used as the loading control. Western blots were developed using NcmECL Ultra (Suzhou, China).

### Immunohistochemistry staining

Immunohistochemistry staining was performed as we described previously [38], in which the tissue slides were stained with a diluted primary antibody against PHF5A (1:50, Novus Biologicals) or Ki-67 (1:2000, Proteintech, Wuhan, China). The staining intensity of PHF5A was scored as 0 (no intensity), 1 (weak intensity), 2 (moderate intensity) and 3 (strong intensity). The degree of staining was scored as 0 (0%), 1 (1-25%), 2 (26-50%), 3 (51-75%) and 4 (76-100%) according to the proportion of immunopositive cancer cells. A histoscore of PHF5A was generated by the sum of staining intensity and degree scores (overall score range, 0–7). HNSCC patients were classified into high expression (4-7) and low expression (0–3) groups based on the histoscore.

### Animal models

Four-week-old female nude mice were obtained from Hunan SJA Laboratory Animal Co. (Changsha, Hunan, China) and maintained under specific pathogen-free conditions. The study was approved by the Animal Ethics Committee of Central South University (No. 202,102,042). For the subcutaneous xenograft model, 1.5 × 10^6^ FaDu cells were injected subcutaneously into the right flank of nude mice. Approximately 4 weeks later, the mice were sacrificed, and tumour samples were harvested. For the metastasis model, 4 × 10^6^ FaDu cells were injected into nude mice via the tail vein. Approximately 6 weeks later, the mice were sacrificed, and lung tissues were fixed and stained with H&E.

### Statistical analysis

Data are shown as the representative results of at least three independent experiments. Statistical analyses of two groups were performed with unpaired Student’s t test (for equal variances) or Mann-Whitney U test (for unequal variances) using SPSS software (version 23.0; SPSS, Inc., Chicago, IL, USA). The chi-squared test was used for statistical analysis of categorical data. *P* < 0.05 was regarded as statistically significant.

## Electronic supplementary material

Below is the link to the electronic supplementary material.

Additional File 1: Supplemental Figure S1. Expression of related proteins in subcutaneous xenograft tissues.

Additional File 2: Supplemental Figure S2. Expression of related proteins in FaDu cells treated with SB203580.

Additional File 3: Supplemental Table S1. Sequences of primers.

Additional File 4: Supplemental Table S2. Correlations between PHF5A expression and clinicopathological parameters in TCGA HNSCC data.

Additional File 5: Supplemental Table S3. Correlations between PHF5A expression and clinicopathological parameters in the HNSCC validation cohort.

## Data Availability

All data generated or analysed during this study are included in this article and its supplementary information files.

## Abbreviations

HNSCC: Head and neck squamous cell carcinoma
TCGA: The Cancer Genome Atlas
snRNP: Small nuclear ribonucleoprotein
PHF5A: PHD finger protein 5 A
MAPK: Mitogen-activated protein kinase
ATCC: American Type Culture Collection
FBS: Fetal bovine serum
siRNA: Small interfering RNA
NC: Negative control
OE: Overexpression
EV: Empty vector
WT: Wild-type
PVDF: Polyvinylidene fluoride
SDS–PAGE: Sodium dodecyl sulphate-polyacrylamide gel electrophoresis
