# Supplementary figures and images for: PHF5A regulates the expression of the DOCK5 variant to promote HNSCC progression through p38 MAPK activation
Source: Biol Direct. 2023 Jul 12;18:39. doi: 10.1186/s13062-023-00396-4 (PMC10337101; doi:10.1186/s13062-023-00396-4)

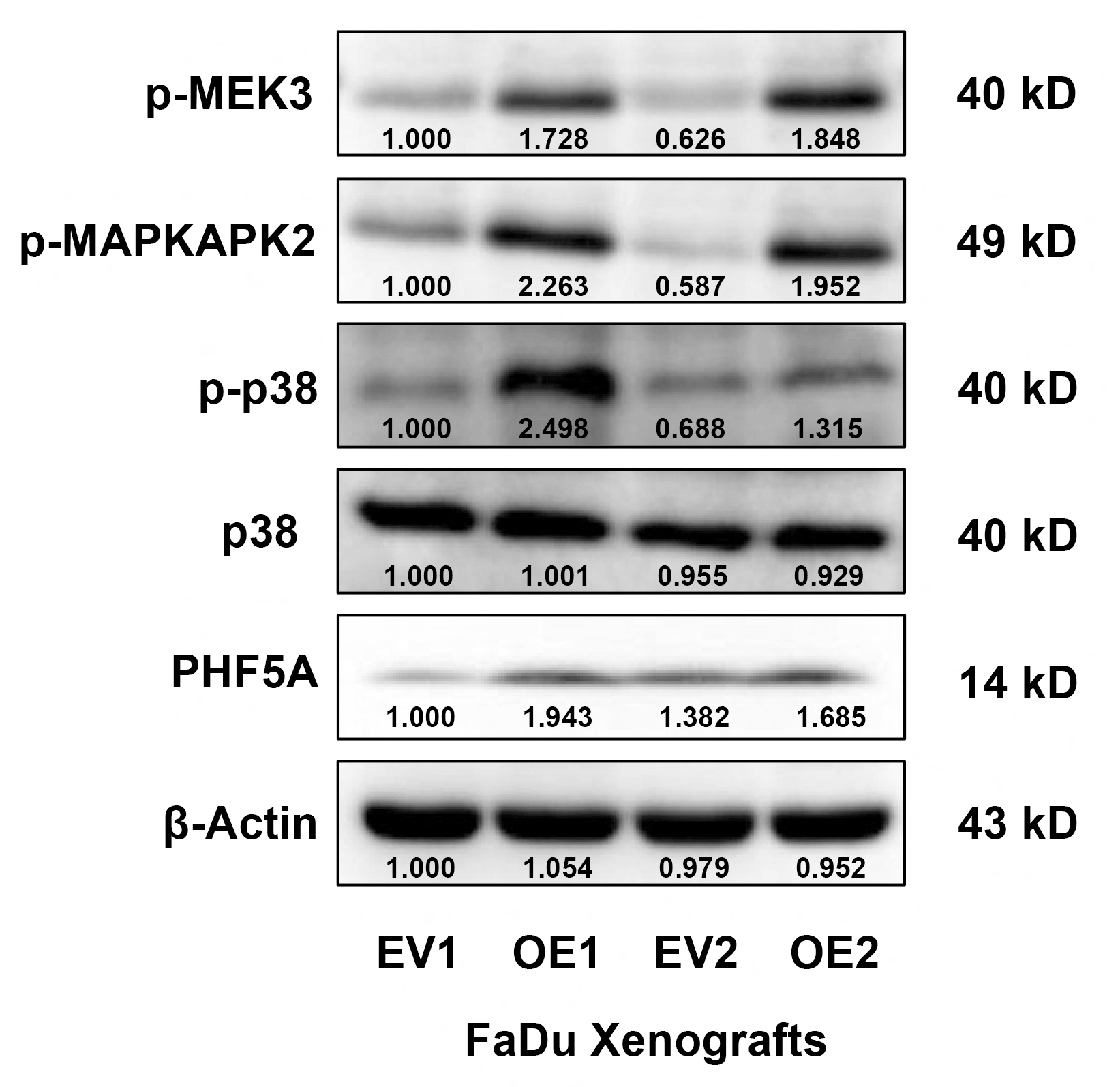

Supplement: Supplementary file 1 — Additional File 1: Supplemental Figure S1. Expression of related proteins in subcutaneous xenograft tissues. [file 13062_2023_396_MOESM1_ESM.png]

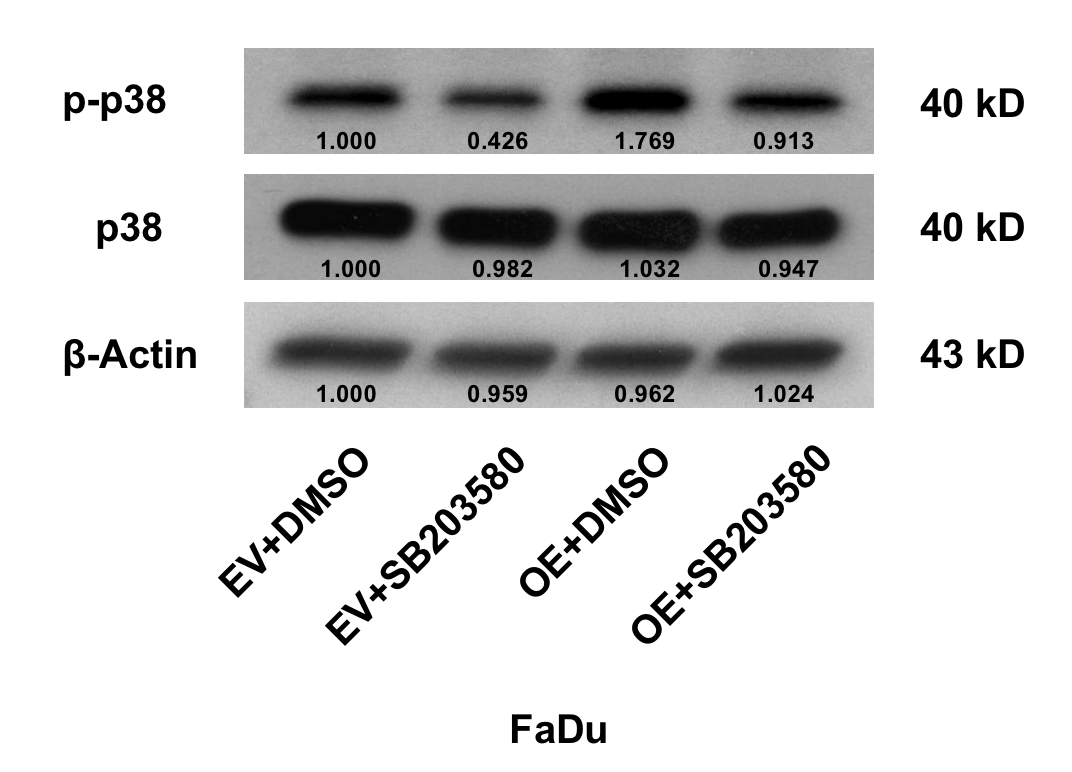

Supplement: Supplementary file 2 — Additional File 2: Supplemental Figure S2. Expression of related proteins in FaDu cells treated with SB203580. [file 13062_2023_396_MOESM2_ESM.png]
